# First-trimester biochemical inflammatory markers (NHR and NLR) as predictors of preeclampsia risk

**DOI:** 10.5937/jomb0-61798

**Published:** 2026-03-17

**Authors:** Qianqian Wang, Xuehui Li, Xiaotao Ma

**Affiliations:** 1 Department of Gynecology, Shunde Hospital Affiliated to Jinan University (Shunde District Second People's Hospital of Foshan City, Shunde District Feng Yaojing Memorial Hospital of Foshan City), Shunde, China; 2 Department of Obstetrics, Shunde Hospital Affiliated to Jinan University (Shunde District Second People's Hospital of Foshan City, Shunde District Feng Yaojing Memorial Hospital of Foshan City), Shunde, China

**Keywords:** preeclampsia, neutrophil-to-HDL ratio, neutrophil-to-lymphocyte ratio, laboratory markers, biochemical indices, early prediction, preeklampsija, odnos neutrofila i HDL, odnos neutrofila i limfocita, laboratorijski markeri, biohemijski indeksi, rana predikcija

## Abstract

**Background:**

Preeclampsia (PE) remains a leading cause of maternal and perinatal morbidity. The early identification of high-risk pregnancies requires accessible and cost-effective laboratory markers. This study aimed to evaluate first-trimester blood count-derived inflammatory indices, with a focus on the neutrophil-to-HDL ratio (NHR) and neutrophil-to-lymphocyte ratio (NLR), for PE risk prediction.

**Methods:**

A retrospective analysis was conducted involving 90 patients with PE and 90 healthy pregnant women. Routine hematological and biochemical parameters were measured using an automated hematology analyzer and an automated biochemical analyzer respectively, and derived indices (NLR, NHR, m H r, SII) were calculated. Independent risk factors were determined through multivariate logistic regression, and a predictive model was validated by receiver operating characteristic (ROC) curve analysis.

**Results:**

PE patients exhibited significantly higher NLR, NHR, and SII levels, compared with controls (all p &lt; 0.001). Multivariate logistic regression identified NLR, NHR, and body mass index (BMI) as independent predictors of PE. The combined model incorporating these factors achieved excellent predictive performance, with an area under the ROC curve of 0.909 (95% CI: 0.866-0.952).

**Conclusions:**

The first-trimester NHR and NLR, combined with BMI, constitute a clinically useful panel for early PE prediction. This readily applicable model provides a valuable alternative to cost-intensive screening methods, particularly in resource-constrained settings. Its implementation could enhance first-trimester risk stratification and guide timely interventions, ultimately contributing to improved maternal and perinatal outcomes.

## Abstract (sr)

**Uvod:**

Preeklampsija (PE) ostaje vodeći uzrok majčinog i perinatalnog morbiditeta. Rana identifikacija trudnoća visokog rizika zahteva pristupačne i isplative laboratorijske markere. Cilj ove studije je bio da se procene inflamatorni indeksi izvedeni iz krvne slike u prvom tromesečju, sa fokusom na odnos neutrofila i HDL (NHR) i odnos neutrofila i limfocita (NLR), za predviđanje rizika od PE.

**Metode:**

Sprovedena je retrospektivna analiza koja je obuhvatila 90 pacijentkinja sa PE i 90 zdravih trudnica. Rutinski hematoloski i biohemijski parametri mereni su korišćenjem automatizovanog hematološkog analizatora, odnosno automatizovanog biohemijskog analizatora, i izračunati su izvedeni indeksi (NLR, NHR, MHR, SII). Nezavisni faktori rizika određeni su multivarijantnom logističkom regresijom, a prediktivni model je validiran analizom ROC krive (receiver operating characteristic).

**Rezultati:**

Pacijenti sa PE pokazali su značajno više nivoe NLR, NHR i SII, u poređenju sa kontrolnom grupom (sve p &lt; 0,001). Multivarijantna logistička regresija identifikovala je NLR, NHR i indeks telesne mase (ITM) kao nezavisne prediktore PE. Kombinovani model koji uključuje ove faktore postigao je odlične prediktivne performanse, sa površinom ispod ROC krive od 0,909 (95% CI: 0,866-0,952).

**Zaključak:**

NHR i NLR u prvom tromesečju, u kombinaciji sa BMI, predstavljaju klinički koristan panel za rano predviđanje PE. Ovaj lako primenljiv model pruža vrednu alternativu skupim metodama skrininga, posebno u uslovima ograničenih resursa. Njegova primena mogla bi poboljšati stratifikaciju rizika u prvom tromesečju i usmeriti blagovremene intervencije, što bi na kraju doprinelo poboljšanju majčinih i perinatalnih ishoda.

## Introduction

Preeclampsia (PE), a multisystem disorder characterized by new-onset hypertension and proteinuria after 20 weeks of gestation, remains a leading cause of maternal and perinatal morbidity and mortality worldwide [1]. Globally, 0.2-9.2% of expectant mothers are impacted, leading to more than 76,000 maternal deaths and 500,000 fetal and newborn deaths each year [2]. PE not only significantly contributes to unfavorable pregnancy outcomes such as early delivery and perinatal death, but also brings long-term health risks to mothers and infants, being closely related to diabetes, chronic kidney disease, and cardiovascular diseases [3][4]. Currently, delivery remains the only definitive treatment for PE [5], often necessitating difficult decisions regarding preterm birth. This underscores the critical importance of early detection. However, because the early stage of PE is generally asymptomatic, early diagnosis remains a clinical challenge.

Traditionally, the UK's National Institute for Health and Care Excellence (NICE) and the American College of Obstetricians and Gynecologists (ACOG) have relied on maternal risk factors to classify pregnant individuals into PE risk categories. Although widely applicable, this approach suffers from low sensitivity [6]. To address this limitation, methods combining mean arterial pressure, uterine artery Doppler pulsatility index, and serum placental growth factor levels have been created for screening. These algorithms significantly improve predictive accuracy. They require specialized equipment (e.g., Doppler ultrasound machines), technical expertise in acquisition and interpretation, and - in the case of PLGF - relatively expensive biomarker assays. These resource demands render such screening logistically challenging and economically prohibitive in many resource- constrained clinical settings, limiting their universal adoption [1][7]. Consequently, there remains a critical need to identify simple, cost-effective, and readily obtainable biomarkers detectable in the first trimester to facilitate early risk stratification and preventive strategies.

The etiology of PE is complex and multifactorial. The core pathophysiological mechanism of PE is the systemic inflammatory response and maternal endothelial dysfunction caused by placental dysfunction [3]. In recent years, various inflammatory factors have been found to be significantly associated with PE and may serve as predictive or diagnostic markers [8]. PE is frequently accompanied by elevated counts of neutrophils, lymphocytes, monocytes, and platelets— a hematological profile associated with increased disease risk [9][10]. In addition, high-density lipoprotein (HDL), as a mediator of inflammation and oxidative stress, plays a significant role in the regulation of inflammation related to PE [11]. Previous studies have indicated that monocyte to HDL ratio (MHR), neutrophil to HDL ratio (NHR), neutrophil to lymphocyte ratio (NLR), and systemic immune-inflammation index (SII) are strongly linked to the state of inflammation in various diseases [12][13]. However, the relationship between these indicators and PE-related inflammation has not been fully elucidated, and their predictive value for PE remains unclear.

Our research focused on exploring the evaluation effect of inflammatory indicators MHR, NLR, NHR and SII derived from blood routine in the first trimester of pregnancy on PE. The development of a predictive model based on these indicators could help identify high-risk individuals early, allowing for intensified monitoring and timely prophylactic measures.

## Materials and methods

### Participants

This retrospective case-control study received approval from the Ethics Committee of Shunde Hospital Affiliated to Jinan University. This study included pregnant women who registered for prenatal care in their first trimester and delivered at Shunde Hospital Affiliated to Jinan University. The pregnant women were divided into two groups: PE (n=90) and control groups (n=90). PE was diagnosed according to the ACOG guidelines [14], Identified as the emergence of hypertension after 20 weeks of gestation (systolic blood pressure ≥140 mmHg and/or diastolic blood pressure ≥90 mmHg), along with either proteinuria (≥0.3 g/ in a 24-hour urine collection or random urine protein ≥1+), or in the absence of proteinuria with the following complications, including thrombocytopenia, liver dysfunction, kidney problems, lung edema, or new cerebral or visual issues. The control group consisted of 90 healthy pregnant women with uncomplicated pregnancies. Written informed consent was obtained from all participants before they were included in the study.

### Inclusion and exclusion criteria

Inclusion criteria were as follows: 1) diagnosis of PE according to ACOG guidelines; 2) singleton pregnancy with live birth; 3) gestational age at delivery ≥25 weeks; 4) maternal age ≥18 years; and 5) provision of written informed consent.

Exclusion criteria included: 1) pre-existing medical conditions, such as hypertension, diabetes, hepatic or renal dysfunction, thyroid disorders, malignant tumors, or cardiovascular diseases; 2) multiple gestation; 3) conception via assisted reproductive technology; 4) history of smoking or alcohol abuse; 5) recent acute or chronic infection; 6) co-existing hemorrhagic or coagulation disorders; and 7) use of antibiotics, anticoagulants, or antiplatelet agents within three months prior to enrollment.

### General data collection

Demographic and clinical data were collected for all participants, including age, pre-pregnancy body mass index (BMI), gestational age at delivery, educational level, gravidity, parity, history of adverse obstetric outcomes (such as unexplained miscarriage, missed abortion, stillbirth, dystocia, fetal malformation, or birth defects), and the presence of other pregnancy complications (intrahepatic cholestasis of pregnancy, gestational anemia, and gestational diabetes mellitus).

### Laboratory measurements

All participants underwent standardized hematological and biochemical testing during the first trimester (gestational age ≤12 weeks). Fasting venous blood samples (5 mL) were collected under aseptic conditions and processed within 2 hours of collection to ensure sample stability. Routine hematological indices, including neutrophil, lymphocyte, monocyte, and platelet counts, were measured using an automated hematology analyzer (Sysmex XN-9000, Sys- mex Corporation, Kobe, Japan). Biochemical asses - sment focused on serum high-density lipoprotein cholesterol (HDL-C), was determined by enzymatic colorimetric assay using the automated biochemistry analyzer cobas c 502 (Roche Diagnostics, Mannheim, Germany). HDL-C is recognized as a key biochemical parameter in lipid metabolism and systemic inflammation, and its integration with hematological indices provides a more comprehensive laboratory profile of maternal immune and metabolic status. Both analyzers were operated with internal quality control procedures performed daily in accordance with the manufacturers' recommendations, thereby ensuring analytical precision and reproducibility.

Based on these primary measurements, several derived inflammatory indices were calculated to capture the balance between cellular and lipid-related components of inflammation:

- Monocyte-to-HDL ratio (MHR) = monocyte count / HDL-C concentration
- Neutrophil-to-lymphocyte ratio (NLR) = neutrophil count / lymphocyte count
- Neutrophil-to-HDL ratio (NHR) = neutrophil count / HDL-C concentration
- Systemic immune-inflammation index (SII) = (platelet count X neutrophil count) / lymphocyte count

These indices have been increasingly applied as laboratory-based inflammatory markers across various clinical contexts, offering low-cost and easily obtainable measures derived from routine blood testing. In the present study, their use in the first trimester was aimed at evaluating their potential role as biochemical indicators for early risk stratification of preeclampsia.

### Statistical analysis

Data were analyzed using the Statistic Package for Social Science (SPSS) 27.0 statistical software (IBM, Armonk, NY, USA). Data normality was assessed through the Shapiro-Wilk tests. Normally distributed data are shown as mean ± SD, and the differences between the two groups were assessed with a student's t-test. Non-normally distributed data are shown as median (interquartile range), and comparison was performed using the Mann-Whitney U test. Count data are displayed as number (n), and group differences were assessed using the chi-square test. In the multivariate logistic regression analysis, all continuous variables, including BMI, MHR, NLR, NHR, and SII, were entered as continuous variables in the model. Variables with notable differences in general characteristics and lab indices between the two groups were included as covariates to find risk factors linked to PE. Based on the clinical risk stratification for PE defined by the ACOG and incorporating the results from the multivariate logistic regression, a rapid risk assessment model was constructed by assigning scores of 0, 1, and 2 to low-, moderate-, and high-risk factors, respectively. The predictive performance evaluation of this model was determined using the receiver operating characteristic (ROC) curve analysis. P<0.05 was considered statistically significant.

## Results

### Comparison of clinical features between PE patients and healthy controls

This study encluded 180 pregnant women, with 90 allocated to the PE group and 90 to the control group. The baseline clinical characteristics are presented in Table 1. No significant differences were observed between the two groups regarding age (mean: 28.28 vs. 27.57, P=0.2169), BMI (mean: 23.71 vs. 23.91, P=0.0937), or gestational age (mean: 37.88 vs. 37.78, P=0.6708). Although the PE group included a higher proportion of women with higher education (college degree or above) and the control group included more women with lower education levels (high school or below), this difference was not statistically significant (P = 0.0969). Additionally, no significant differences were found in gravidity (≤2/>2, P=0.6715), parity (primiparous/multiparous, P=0.7245), or history of adverse pregnancy outcomes (yes/no, P=0.2731). The incidence of pregnancy-related complications, including intrahepatic cholestasis of pregnancy (P=0.7324) and gestational anemia (P=0.3668), did not differ significantly between the groups. Nevertheless, The incidence of gestational diabetes mellitus was significantly elevated in the PE group compared to the control group (17 vs. 10, P=0.0283).

**Table 1:** Comparison of various indicators between two groups of samples. Note: Data are presented as mean ± standard deviation, median (interquartile range), or n (%).

Variable	Control (n=90)	PE (n=90)	Statistical test	Test statistic	P-value
Age	27.57±3.43	28.28±3.21	t-test	1.239	0.2169
BMI	23.91±6.52	23.71±4.68	t-test	1.685	0.0937
Gestational week	37.78±2.47	37.88±3.41	t-test	0.4257	0.6708
Educational level			Chi-square test	1.660	0.0969
High school and below	76	67			
College and Above	14	23			
Gravidity			Chi-square test	0.4241	0.6715
≤2	76	78			
2	14	12			
Parity			Chi-square test	0.3525	0.7245
1	70	68			
≥2	20	22			
Adverse pregnant<br>production history			Chi-square test	1.096	0.2731
No	74	68			
Yes	16	22			
Intrahepatic cholestasis<br>of pregnancy			Chi-square test	0.3420	0.7324
No	86	85			
Yes	4	5			
gestational anemia			Chi-square test	0.3885	0.3668
No	87	86			
Yes	3	4			
Pregnancy associated <br>with diabetes			Chi-square test	2.193	0.0283
No	83	73			
Yes	7	17			
MHR	0.40±0.06	0.43±0.07	t-test	3.333	0.001
NLR	3.52±0.51	4.35±0.37	t-test	9.226	P<0.0001
NHR	3.76 (3.30 4.31)	4.96 (4.55 5.62)	Mann-Whitney U test	1.255	P<0.0001
SII	857 (746 969)	1085 (966 1192)	Mann-Whitney U test	219.1	P<0.0001

Subsequently, blood count and biochemical parameters were measured using an automated hematology analyzer and an automatic biochemistry analyzer, respectively, and the derived indices MHR, NLR, NHR, and SII were calculated. The results showed that MHR (mean: 0.43 vs. 0.40, P=0.001), NLR (mean: 4.35 vs. 3.52, P<0.0001), NHR (median: 4.96 vs. 3.76, P<0.0001), and SII (median: 1085 vs. 857, P<0.0001) were markedly increased in the PE group relative to the controls (Table 1).

### Evaluation of PE-associated risk factors

Variables with P<0.1 in univariate analysis (Table 1) were selected as independent variables and assigned values as detailed in Table 2. PE was coded as 1 (present) or 0 (absent). Educational level was assigned a value of 2 for high education (college degree or above) and 1 for low education (high school or below). Gestational diabetes mellitus was assigned as 2 (present) or 1 (absent). Each continuous variable was entered as such in the analysis. To investigate the connection between these factors and PE risk, multivariate logistic regression was utilized. The results indicated that NLR (Wald 8.597, P=0.003, 95%CI: 1.959-29.524), NHR (Wald 9.448, P=0.002, 95%CI: 1.639-9.339), and BMI (Wald 9.068, P=0.003, 95%CI:1.097-1.546) were positively correlated with PE (Table 3). Specifically, each 1-unit increase in NHR was associated with a 3.913-fold increase in the risk of developing PE, after adjusting for other covariates in the model. The relatively high odds ratio for NHR may reflect its strong association with PE pathophysiology, integrating both inflammatory and lipid metabolic pathways. In contrast, GDM, MHR, SII, and educational level showed no significant association with PE risk (all P > 0.0; Table 3).

**Table 2:** Assignment instructions.

Variable	Variable type	Assignment instructions
Preeclampsia	Categorical variable	"0"=No "1"=Yes
BMI	Continuous variable	-
Educational level	Categorical variable	"1"=High school and below "2"=College and AboveGravidity
Pregnancy associated with diabetes	Categorical variable	"1"=No "2"=Yes
MHR	Continuous variable	-
NLR	Continuous variable	-
NHR	Continuous variable	-
SII	Continuous variable	-

**Table 3:** Logistic multiple regression analysis of PE occurrence.

Variable	B	SE	wald	P-value	Exp (B)	EXP (B) 95% <br>confidence interval	
						lower limit	upper limit
Pregnancy associated <br>with diabetes	0.913	0.716	1.625	0.202	2.493	0.612	10.151
MHR	1.476	4.319	0.117	0.733	4.377	0.001	20792.064
NLR	2.029	0.692	8.597	0.003	7.606	1.959	29.524
NHR	1.364	0.444	9.448	0.002	3.913	1.639	9.339
SII	0	0.002	0.012	0.912	1	0.996	1.004
BMI	0.264	0.088	9.068	0.003	1.302	1.097	1.546
Educational level	-0.556	0.494	1.268	0.26	0.573	0.218	1.51
Constant	-20.74	3.85	29.023	0	0		

### Construction and evaluation of the model for assessing PE risk

Based on the multivariate logistic regression results, NLR, NHR, and BMI were incorporated into the risk assessment model. In accordance with ACOG guidelines, BMI was recognized as a risk factor for PE, and a BMI >25 kg/m^2^ was assigned 1 point. As current guidelines do not specify NLR and NHR as established risk factors for PE, their cut-off values were defined based on the study measurements. Specifically, based on the distribution of individual values and group means of NLR and NHR between the control and PE groups, we determined that cut-off values of NLR ≥ 3.92 and NHR ≥ 4.08 effectively discriminated PE patients from controls. The point assignments were determined according to their odds ratios [Exp(B)]. The Exp(B) value for NLR was 7.606, which is close to 2^3^ (equals 8), thus it was assigned 3 points. The Exp(B) value for NHR was 3.913, approximating 2^2^ (equals 4), and was consequently assigned 2 points. Taken together, NHR ≥4.08 was assigned 2 points, and an NLR ≥3.92 was assigned 3 points. Consequently, the total risk score spanned from 0 to 6 (Table 4).

**Table 4:** Quick assessment checklist for PE.

Risk factor	Score
BMI >25 kg/m^2^	1
NHR ≥4.08	2
NLR ≥3.92	3
Total score range	0-6

**Figure 1:**
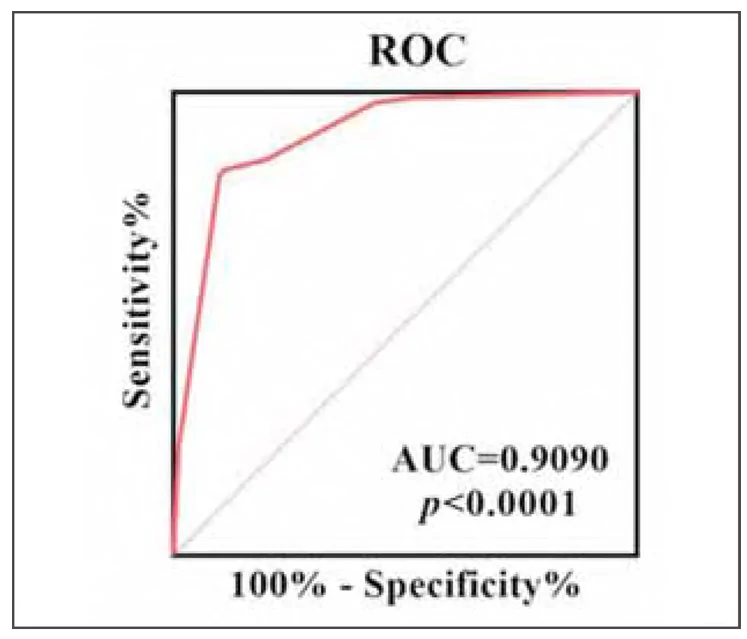
The ROC curve of the PE risk assessment model. AUC=0.9090, 95%CI: 0.8656-0.9524, Cutoff value=3.5, Sensitivity=0.833, Specificity=0.889, Youden index=0.722.

Then, the predictive performance of the model was analyzed using ROC curve analysis. The area under the curve (AUC) was 0.9090 (95% CI: 0.8656-0.9524, P < 0.0001). The model determined a cutoff value of 3.5, corresponding to a sensitivity of 0.833, specificity of 0.889, and a Youden's index measuring 0.722 (Figure 1).

## Discussion

This study developed and validated a straightforward laboratory-based tool for assessing preeclampsia (PE) risk using first-trimester blood count-derived inflammatory indices combined with maternal characteristics. The central finding is that a model incorporating neutrophil-to-lymphocyte ratio (NLR), neutrophil-to-HDL ratio (NHR), and body mass index (BMI) demonstrated excellent predictive performance, underscoring the potential of routine laboratory parameters as early biochemical markers for high-risk pregnancies.

Early recognition of women at risk of PE is essential for timely intervention, and laboratory medicine plays a pivotal role in this process. Aberrant inflammatory responses are closely linked to PE pathophysiology, as immune dysregulation during the first trimester contributes to later disease development [15]. Several blood-derived indices, including monocyte-to-HDL ratio (MHR), NHR, NLR, and systemic immune-inflammation index (SII), have been evaluated as inflammatory markers across different diseases, such as Parkinson's disease [16], obesity [17], and diabetes [18]. Their application to pregnancy-related disorders highlights the translational value of routine hematological and biochemical tests in predicting disease risk, although prior conclusions regarding PE remain inconsistent.

Our data demonstrated that MHR, NHR, NLR, and SII were significantly elevated in PE patients, in line with the underlying inflammatory pathophysiology. Importantly, only NLR and NHR emerged as independent predictors. NLR, a systemic inflammation marker, has been reported to correlate with adverse outcomes in PE [9]. Our findings are consistent with Kang et al. [19], who observed increased NLR in severe cases of PE, supporting its role as a reliable laboratory indicator. By contrast, Cui et al. [20] noted reduced NLR values in PE patients, which might be attributed to differences in disease severity spectrum or the timing of blood sampling relative to disease onset. However, Seyhanli et al. [21] suggested that first-trimester NLR lacks predictive value. These contradictory conclusions may arise from several methodological and population-specific factors, including include potential differences in genetic background, prevalence of comorbidities, the specific gestational week within the first trimester when blood was drawn, and the statistical approaches used for determining predictive cut-offs. These discrepancies likely reflect differences in study design, cohort characteristics, and analytical thresholds, highlighting the need for standardized laboratory methodologies.

The incorporation of NHR adds a novel biochemical perspective by linking inflammatory responses with lipid metabolism. PE is an inflammation-related disease. Neutrophils can cause systemic inflammation and oxidative stress, leading to endothelial damage [22]. The increase in their number is closely related to PE [23]. On the contrary, HDL cholesterol is known for anti-inflammatory and antioxidant functions that protect endothelial health [24]. Reduced HDL levels have been associated with PE development [25], which may reflect a diminished capacity to counteract placental oxidative stress, thereby exacerbating endothelial dysfunction. The NHR thus synergistically captures both the provocation (inflammation) and the impaired protection (dyslipidemia) [26], offering a more integrated biomarker than inflammatory indices alone. Our results confirms that NHR is significantly elevated in patients with PE. This finding aligns with a recent report suggesting NHR as a promising early biomarker for PE [27]. Together with BMI - an established metabolic and inflammatory risk factor for PE [28][29] - these indices provide a cohesive framework for early risk assessment rooted in the interplay of inflammation and metabolic dysregulation.

By integrating NLR, NHR, and BMI, we constructed a predictive model with strong diagnostic accuracy (AUC = 0.9090). Compared with single markers, this multifactorial approach provides a more complete biochemical profile of maternal inflammation and metabolism, thereby improving sensitivity and specificity. Importantly, this model utilizes simple, inexpensive, and widely available laboratory tests, making it highly applicable to routine prenatal screening. In clinical practice, such a model could stratify patients into different risk categories, enabling tailored monitoring strategies and prophylactic interventions such as low-dose aspirin [30].

Nonetheless, several limitations should be acknowledged. The single-center, case-control design may limit generalizability, and the relatively small sample size could affect the stability of the estimates. Moreover, the thresholds for NLR and NHR were derived from this cohort and require external validation. Future multicenter prospective studies are warranted to confirm these laboratory cutoffs and ensure broader applicability.

## Conclusion

In summary, this study demonstrates the utility of routine laboratory markers - particularly NLR and NHR - as reliable biochemical indicators for early PE risk assessment. When combined with BMI, these indices form a practical, low-cost, and highly accurate predictive model. By leveraging hematological and biochemical measurements that are readily accessible in clinical laboratories, this approach supports the role of laboratory medicine in advancing accessible and informative clinical decision-making. Further prospective, multicenter validation is essential to establish standardized thresholds and expand the application of this model in routine prenatal care.

## Dodatak

### Funding

This work was supported by the Self-funded scientific and technological innovation projects of Foshan City in 2024 (project number: 2420001004608) project name: blood routine derivative inflammation index based on the early stages of pregnancy express build touting the forecast effect of preeclampsia risk assessment test.

### Conflict of interest statement

All the authors declare that they have no conflict of interest in this work.
